# An Estimation of the Requirements of the Standardized Ileal Digestible Tryptophan, Valine, Isoleucine and Methionine on Young Pigs’ (Up to 50 kg) Feed Efficiency: A Meta-Regression Analysis

**DOI:** 10.3390/ani14192884

**Published:** 2024-10-07

**Authors:** Byungho Chae, Junior Isaac Celestin Poaty Ditengou, A-Leum Lee, Jisoo Tak, Inhyeok Cheon, Nag-Jin Choi

**Affiliations:** 1 Department of Animal Science, Jeonbuk National University, Jeonju 54896, Republic of Korea; byungho721@jbnu.ac.kr (B.C.); celestinisaacjunior10@jbnu.ac.kr (J.I.C.P.D.); cih6770@jbnu.ac.kr (I.C.); 2 CJ Cheiljedang, Seoul 04560, Republic of Korea; aleum.lee1@cj.net (A.-L.L.); js.tak@cj.net (J.T.)

**Keywords:** swine, SID, amino acid, ratio, meta-regression

## Abstract

**Simple Summary:**

A new estimate of swine needs in terms of amino acids seems necessary according to the factors influencing them and the current conditions within the pig industry. Using a meta-regression analysis, we found that the requirements of tryptophan, valine, isoleucine, and methionine were superior to previous recommendations regardless of the regression models and the animals’ growth phases. Among the regression models used, two were selected as estimations of amino acid requirements for pigs under challenged conditions, whereas another one was chosen to estimate the amino acid requirements of genetically improved pigs under a modern housing system. These outcomes might serve as a foundation to build new standards in the assessment of pig requirements.

**Abstract:**

Currently, the NRC amino acid (AA) requirements for pigs published in 2012 are used as a reference in variable swine industries. However, recent results in several articles suggest that the standardized ileal digestible (SID) AA–lysine (Lys) ratio significantly evolved over the last two decades, while some publications report inconsistent outcomes. Therefore, the present study used a meta-regression analysis to assess the relative ratio to lysine to maximize the feed efficiency of four essential amino acids (tryptophan, valine, isoleucine, and methionine) in pig diets. According to the PRISMA guidelines, articles examining the target AA requirement using a basal diet supplemented with varying levels of crystalline AA (tryptophan, valine, isoleucine, or methionine) were identified across Scopus, PubMed, and Science Direct. As a result, 23, 22, 16, and 9 articles using tryptophan, valine, isoleucine, and methionine were selected and categorized into experiments for inclusion in our meta-analysis. The results suggested that the requirements of tryptophan, valine, isoleucine, and methionine in our meta-regression analysis were superior to NRC recommendations, regardless of the regression models and the growth phases with significant RSQ values (RSQ ≈ 1). Also, the QUAD and CLP regression models emphasized higher requirements than the LP model for the great majority of amino acids and growth phases. The results of the QUAD and CLP models were selected as estimations of the amino acid requirements for pigs under challenged conditions, whereas the LP model was chosen to estimate the amino acid requirements of genetically improved pigs under a modern housing system. The results of this meta-regression analysis could be used to refresh the information on the NRC amino acids (AA) requirements for swine.

## 1. Introduction

Nitrogen (N), excessively excreted into natural environments by pigs raised in modern farms, has a detrimental impact through its contribution to the acidification and eutrophication of sensitive ecosystems and odor emissions [1]. The reduction in crude protein (CP) content in swine diets has proven to decrease these negative effects by improving nitrogen (N) utilization efficiency, with beneficial outputs on both animal performance and health as well as reduced environmental impact [2,3]. However, this feeding strategy should be combined with an appropriate provision of amino acids (AA) in terms of quantity and quality to meet the animals’ genetic potential for protein deposition [4,5]. Until recently, the NRC [6] has been the reference for providing appropriate amino acid requirements in a pig’s diet with low levels of CP. However, recent studies suggested that the standardized ileal digestible (SID) AA–lysine (Lys) ratio significantly evolved in the last two decades, while some publications showed inconsistent outcomes [7,8,9,10]. Moreover, the NRC requirements are the condensed results from only six studies, of which pig body weight did not surpass 33 kg [11]. Hence, a more accurate investigation considering every factor influencing the amino acid requirements (especially the limited amino acids) in pig nutrition seems to be necessary. In this context, aggregating the findings across all published papers is crucial to ultimately evaluate the requirements of the target amino acid in swine nutrition. Utilizing meta-regression analysis, a statistical tool to obtain an unbiased evaluation from multiple articles [12], is an appropriate approach to achieve these objectives. Indeed, meta-regression analysis can appropriately combine study findings and summarize the genuine impact of an intervention by drawing plots with statistical models, considering the sources of heterogeneity among studies and increasing the sample size.

Among the essential amino acids, tryptophan (Trp), valine (Val), isoleucine (Ile), and methionine (Met) play important roles in several biological functions in pig metabolism. Trp is involved in the regulation of the immune response, voluntary feed intake, and the synthesis of serotonin [13,14,15]. Val and Ile belong to the group of essential branched-chain AA (BCAA), which are interdependent amino acids influencing the inclusion levels of other limiting AA. Furthermore, Val deficiency is known to decrease the feed intake within 1 h after meal ingestion in the pig’s growing stage [16]. Concerning Met, it is recognized as a component of the sulfur amino acids (SAAs), essential not only for protein accretion but also for producing cytokines, glutathione (GSH), and acute phase proteins (APPs) [17]. In addition to its functions in protein synthesis, Met plays key roles in biological functions (e.g., cellular redox function, cell survival and proliferation) and protection against intestinal epithelial growth in young pigs [17]. Based on their importance and the fact that they have not been sufficiently investigated, additional studies are needed to estimate their requirements in pig nutrition.

In the process of assessing the requirements of these AAs (Trp, Val, Ile, and Met), numerous statistical models have been developed [18], but the choice of method depends essentially on the species of interest, application facilities, and the best fit of the data. The linear broken-line model, for instance, assumes a constant efficiency of AA utilization below the breakpoint, where the efficiency is a linear function of the nutrient supplementation under the plateau [19]. However, linear broken-line regression supposes that the response to a nutrient dose is linear, while it is generally not linear because of the rate of change due to the reduction in nutrient dose as the nutrient dose approaches its requirement [20]. For some datasets, a linear broken-line model will underestimate the requirement, in that it represents the average animal in the population [21]. The quadratic model is easy to fit to data (only three input levels are needed for quadratic responses) and can easily fit the increases and decreases in performance [22]. However, the great majority of nutritional outcomes are known to have a plateau or “safe” level between the requirement for response levels and levels inducing negative effects, whereas a quadratic model does not characterize the nutrient response data [22]. Considering the diverse impacts of the regression models outlined in the previous statements, a meta-regression analysis was conducted to investigate the requirements of the limited amino acids of interest (Trp, Val, Ile, and Met) in pigs using the linear-plateau (LP), curvilinear-plateau (CLP), and quadratic (QUAD) regression models. The hypothesis tested in this study was that the requirement of the SID Trp, Val, Ile, and Met will be greater than the levels recommended in NRC [6] due to improved genetic potentials and an increased number of studies testing amino acid requirements under variable factors, such as disease challenge models and antagonism between amino acids.

## 2. Materials and Methods

### 2.1. Literature Search and Study Selection

The literature search was conducted according to the Preferred Reporting Items for Systematic Reviews and Meta-Analyses (PRISMA) updated guidelines [23]. Research papers were sourced from various databases including Scopus (https://www.scopus.com/; accessed on 11 April 2023), PubMed (https://pubmed.ncbi.nlm.nih.gov/; accessed on 11 April 2023), and ScienceDirect (https://www.sciencedirect.com/; accessed on 11 April 2023). The keywords used to search for the eligible papers were the name of the target AA, pig, and its synonyms (piglet, hog, barrow, gilt, and swine). The study selection was made through the record of articles containing the keywords in their titles and abstracts. Then, a full-text screening was processed using the same keyword to collect the final list of eligible papers. The articles retained to conduct this meta-regression analysis were peer-reviewed publications between 2000 and 2023.

### 2.2. Inclusion and Exclusion Criteria

The articles identified from the three databases were compiled in EndNote (version 20.6), and duplicates were automatically eliminated. The remaining records were independently screened by two reviewers based on the following criteria: in vivo experiments published in English (1) investigating the target AA requirement using a basal diet supplemented with varying levels of crystalline AA; (2) research providing a standardized ileal digestible (SID) AA profile; (3) studies using pigs weighing less than 50 kg as experimental animals; (4) assessments of the effects of different target AA requirements on growth parameters (body weight [BW], average daily gain [ADG], average daily feed intake [ADFI], and gain to feed intake ratio [G: F]). Conversely, experiments using unique basal diets were excluded.

### 2.3. Data Extraction and Cleansing

The studies were categorized into experimental groups based on various factors, including the type of experiments conducted, the basal diets used, and the experimental phase. The characteristics of each experimental group, such as authors, publication year, the title of the paper, basal diet, crude protein (%DM), target SID AA (%DM), SID Lys (%DM), target SID AA: Lys ratio, initial BW (kg), final BW (kg), ADG (g/day), ADFI (g/day), and G: F ratio were extracted from each study. To further refine our analysis and detect any outliers, we used quantile ranges of the target SID AA: Lys ratio. The classification by BW for the target SID AA: Lys ratio requirements considered the stage of pig growth followed by National Research Council (NRC, 2012) guidelines: Phase 1 (BW < 11 kg), Phase 2 (11 kg ≤ BW < 25 kg), Phase 3 (25 kg ≤ BW < 50 kg). During our exploratory data analysis, we observed that in certain treatment groups, the response decreased with an increase in the dose, which was contrary to the expected biological or pharmacological response pattern. This indicated the possible presence of confounding factors or experimental errors, potentially obscuring the true dose–response relationship. To preserve the integrity and reliability of our models, these data points were excluded from the final analysis. This precaution is in alignment with the established best practices in data analysis, where data that deviate from expected patterns are thoroughly evaluated and, if deemed necessary, omitted to avoid drawing incorrect conclusions. Focusing on data that display a consistent and interpretable response pattern, we sought to provide a more accurate and robust analysis of the dose–response relationship. This, in turn, enhances the validity of our results and ensures they accurately represent the biological mechanisms at play. We included only the experiments with at least two levels of the target SID AA: Lys ratio to guarantee a valid dose–response analysis.

### 2.4. Statistical Analysis

All statistical analyses were conducted using R software (R Core Team, 2023). The requirements for the target SID AA: Lys ratio to improve G: F in pigs were analyzed. Trials were categorized by the basal diet(s) reported in each experiment. The highest response within each group was set to 100%, and the remaining responses were standardized relative to the highest value observed. The response of pigs to an increase in target AA was modeled using the linear-plateau (LP), curvilinear-plateau (CLP), and quadratic (QUAD) regression models:

(1) $$\begin{array}{cc} \mathrm{LP}: & y\;=\;ax\;+\;b\;\mathrm{for}\;x<BP\;;\;y\;=\;a\times BP\;+\;b\;\mathrm{for}\;x\;\geq BP\\ & y\;=\;\mathrm{Relative}\;\mathrm{response},\;\%\\ & a\;=\;\mathrm{The}\;\mathrm{linear}\;\mathrm{slope}\\ & b\;=\;\mathrm{The}\;\mathrm{intercept}\\ & BP\;=\;\mathrm{The}\;\mathrm{minimum}\;\mathrm{SID}\;\mathrm{AA}:\;\mathrm{Lys}\;\mathrm{ratio}\;\mathrm{required}\;\mathrm{to}\;\mathrm{reach}\;\mathrm{the}\;\mathrm{plateau}\;\mathrm{value}. \end{array}$$

(2) $$\begin{array}{cc} \mathrm{CLP}: & \;y\;=\;ax^{2}\;+\;bx\;+\;c\;\mathrm{for}\;x<BP\;;\;y\;=\;a\times BP^{2}\;+\;b\times BP+c\;\mathrm{for}\;x\geq BP\\ & y\;=\;\mathrm{Relative}\;\mathrm{response},\;\%\\ & a\;=\;\mathrm{The}\;\mathrm{quadratic}\;\mathrm{coefficient}\\ & b\;=\;\mathrm{The}\;\mathrm{linear}\;\mathrm{slope}\\ & c\;=\;\mathrm{The}\;\mathrm{intercept}\\ & BP\;=\;\mathrm{The}\;\mathrm{minimum}\;\mathrm{SID}\;\mathrm{AA}:\;\mathrm{Lys}\;\mathrm{ratio}\;\mathrm{required}\;\mathrm{to}\;\mathrm{reach}\;\mathrm{the}\;\mathrm{plateau}\;\mathrm{value}. \end{array}$$

(3) $$\begin{array}{cc} \mathrm{QUAD}: & y\;=\;ax^{2}\;+\;bx\;+\;c\\ & y\;=\;\mathrm{Relative}\;\mathrm{response},\;\%\\ & a\;=\;\mathrm{The}\;\mathrm{quadratic}\;\mathrm{coefficient}\\ & b\;=\;\mathrm{The}\;\mathrm{linear}\;\mathrm{slope}\\ & c\;=\;\mathrm{The}\;\mathrm{intercept} \end{array}$$

The LP model identifies the optimal AA level for maximal growth in pigs until a plateau is reached. The CLP model provides deeper insights by analyzing both the growth phase and the plateau, identifying the 95% breakpoint (BP) where additional AA no longer improves performance. The QUAD model highlights the critical 95% peak value, indicating when the effectiveness of AA supplementation begins to decline, thus demonstrating the diminishing returns of excessive supplementation. The adequacy of these models was primarily confirmed by evaluating the adjusted R-squared value (RSQ), which quantifies the proportion of variance in the dependent variable explained by the independent variables, adjusted for the number of predictors.

## 3. Results

### 3.1. Tryptophan Requirements

#### 3.1.1. Dataset of Tryptophan

**Figure 1:**
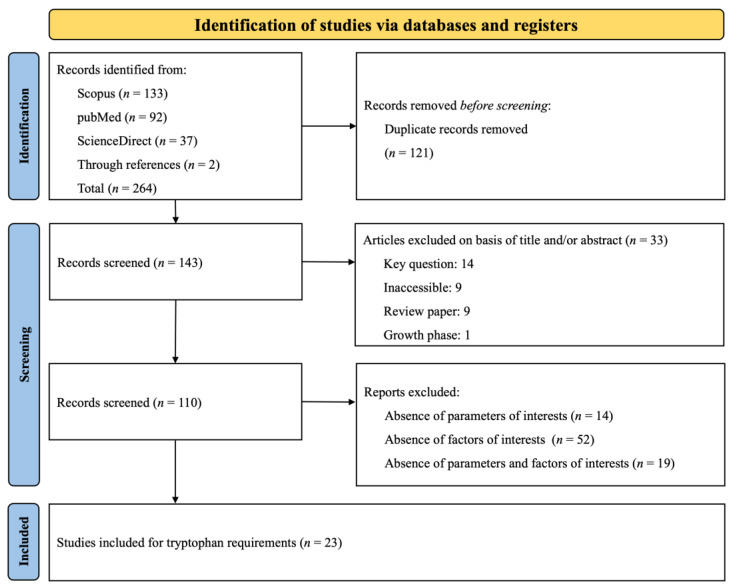
The systematic literature search selection process. The PRISMA diagram details the applied search and selection process for Trp.

The PRISMA flow diagram, presented in Figure 1, summarizes the search strategy employed to investigate Trp requirements. A total of 264 citations were identified across various databases (Scopus: 133, PubMed: 92, ScienceDirect: 37), and 2 additional citations were obtained through reference lists. During the screening process, duplicates and studies that did not meet the specific inclusion criteria were removed. Ultimately, 23 papers were selected for data extraction. Given that some studies included multiple treatments (involving various experiments, basal diets, and phases), these 23 papers were categorized into several distinct experiments according to the phase (Phase 1: *n* = 37; Phase 2: *n* = 23; Phase 3: *n* = 35). An experiment was defined as the implementation of a basal diet associated with one or more Trp inclusions, expressed as the SID Trp: Lys ratio. The datasets and experimental conditions of the 23 studies, recorded after complete screening, are presented in Table 1. The table lists the authors and year of publication and categorizes each study according to the growth phase of the subjects. It also details the range of SID Trp: Lys ratio and the range of SID Lys and provides remarks on specific challenge studies. The compilation of research included in the table spans from 2003 to 2022.

#### 3.1.2. Estimation of the SID Trp: Lys Ratio Requirement

**Figure 2:**
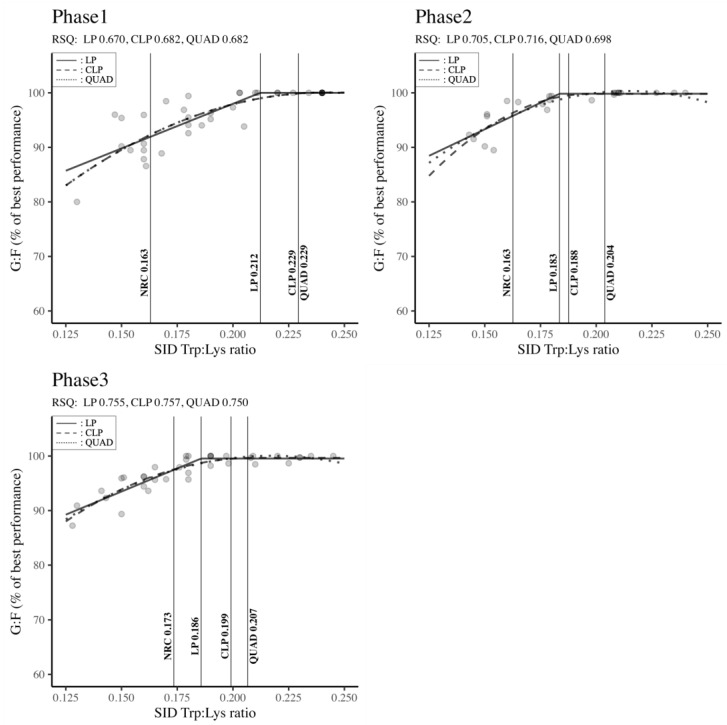
Standardized ileal digestible tryptophan–lysine ratio by body weight classification to maximize feed efficiency in pigs. Phase 1 (BW < 11 kg), Phase 2 (11 kg ≤ BW < 25 kg), Phase 3 (25 kg ≤ BW < 50 kg). RSQ: adjusted R-squared value, LP: linear-plateau model, CLP: curvilinear-plateau model, QUAD: quadratic model. G: F: feed efficiency, SID: standardized ileal digestible.

The requirements of SID Trp: Lys ratio by body weight classification (Phase 1, Phase 2, and Phase 3) to maximize G: F are reported in Figure 2. The Trp: Lys ratio according to the LP, CLP and QUAD models were all superior to the NRC [6] requirements regardless of the growth phase. In Phase 1, the results presented in the diagram (Figure 2) show that the CLP and QUAD models, with equal values, highlighted higher requirements in terms of the SID Trp: Lys ratio than the LP model and the NRC requirements (QUAD: 0.229 = CLP: 0.229 > LP: 0.212 > NRC: 0.163). Similarly, the QUAD model induced the highest requirements, successively followed by the CLP, LP, and NRC outputs in Phase 2 (QUAD: 0.204 > CLP: 0.188 > LP: 0.183 > NRC: 0.163) and Phase 3 (QUAD: 0.207 > CLP: 0.199 > LP: 0.186 > NRC: 0.173). Independent of the phase and regression model, significant RSQ values (RSQ ≈ 1) wavering between 0.670 and 0.757 indicated that the estimation of the SID Trp: Lys ratio requirements was explained by the independent variables in the model.

### 3.2. Valine Requirements

#### 3.2.1. Dataset of Valine

**Figure 3:**
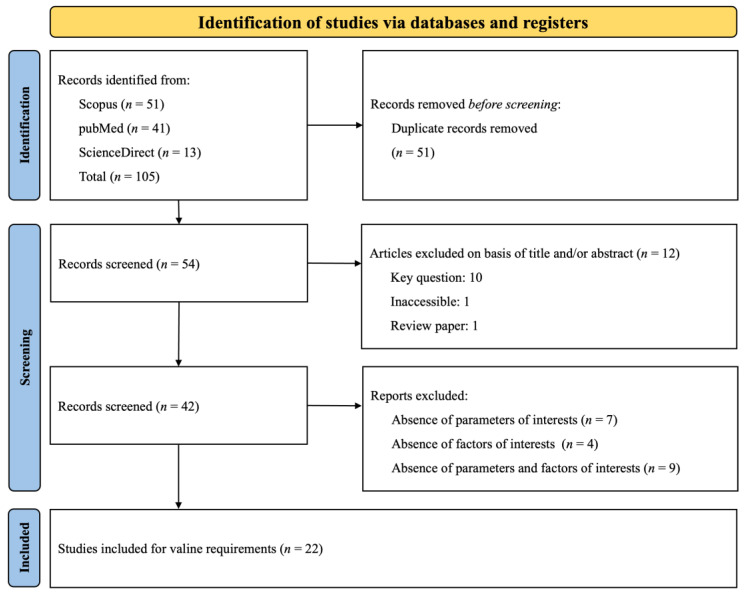
The systematic literature search selection process. The PRISMA diagram details the applied search and selection process for Val.

The PRISMA flow chart depicted in Figure 3 encapsulates the methodology adopted for researching Val requirements. We identified 105 citations from a range of databases: Scopus yielded 51, PubMed 41, and ScienceDirect contributed 13 references. Throughout the vetting process, we excluded duplicate entries and studies that fell outside our predefined selection criteria. Finally, 22 articles were earmarked for in-depth data extraction. Owing to the presence of multifaceted treatment protocols in some studies, encompassing a variety of experiments, basal diets, and growth phases, these 22 articles were organized into multiple unique experimental groups depending on the phase (Phase 1: *n* = 30; Phase 2: *n* = 39; Phase 3: *n* = 21). We delineated an experiment as any application of a basal diet complemented by one or more valine additions, quantified by the SID Val: Lys ratio. The accumulated data and the specifics of the experimental setups for the 22 scrutinized studies are presented in Table 2. This table highlights the growth phase categorization for the studies, the spectrum of the SID Val: Lys ratios, the SID Lys ranges, and annotations regarding interactions with branched-chain amino acids (BCAAs). The compilation of studies spans a publication period from 2009 to 2023.

#### 3.2.2. Estimation of the SID Val: Lys Ratio Requirement

**Figure 4:**
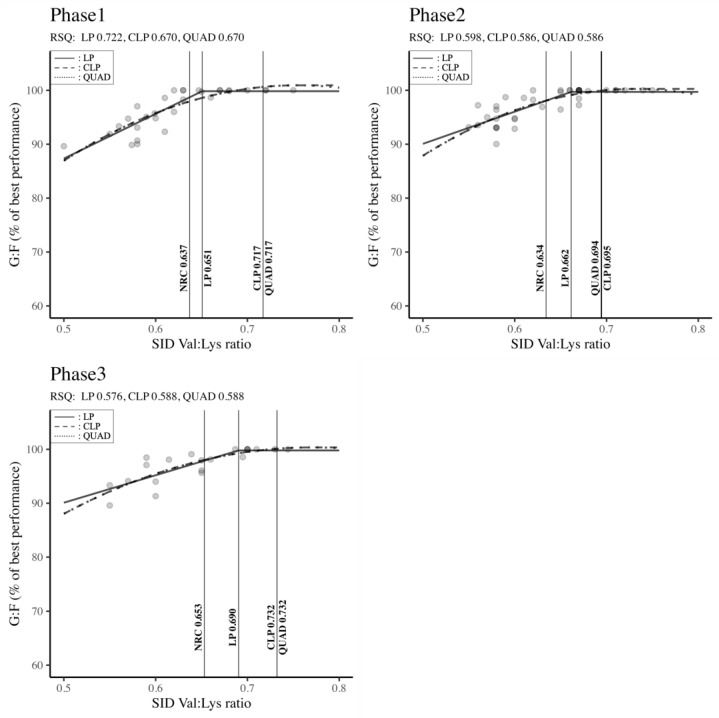
Standardized ileal digestible valine—lysine ratio by body weight classification to maximize feed efficiency in pigs. Phase 1 (BW < 11 kg), Phase 2 (11 kg ≤ BW < 25 kg), Phase 3 (25 kg ≤ BW < 50 kg). RSQ: adjusted R-squared value, LP: linear-plateau model, CLP: curvilinear-plateau model, QUAD: quadratic model. G: F: feed efficiency, SID: standardized ileal digestible.

Figure 4 showed the optimal SID Val: Lys ratio requirements across different body weight classifications (Phase 1, Phase 2, and Phase 3) to maximize G: F. Irrespective of the growth phase, the Val: Lys ratios determined by the LP, CLP, and QUAD models surpassed the NRC (2012) standards. Notably, in Phase 1 and Phase 3, both the CLP and QUAD models exhibited higher Val: Lys ratio requirements compared to the LP model and NRC recommendations (Phase 1: QUAD: 0.717 = CLP: 0.717 > LP: 0.651 > NRC: 0.637; Phase 3: QUAD: 0.732 = CLP: 0.732 > LP: 0.690 > NRC: 0.653). In Phase 2, the CLP model indicated the highest requirements, followed by QUAD, LP, and NRC (CLP: 0.695 > QUAD: 0.694 > LP: 0.662 > NRC: 0.634). Across all phases and regression models, robust RSQ values (RSQ ≈ 1), ranging from 0.576 to 0.722, affirmed that the estimation of SID Val: Lys ratio requirements was explained well by the independent variables within the model.

### 3.3. Isoleucine Requirements

#### 3.3.1. Dataset of Isoleucine

**Figure 5:**
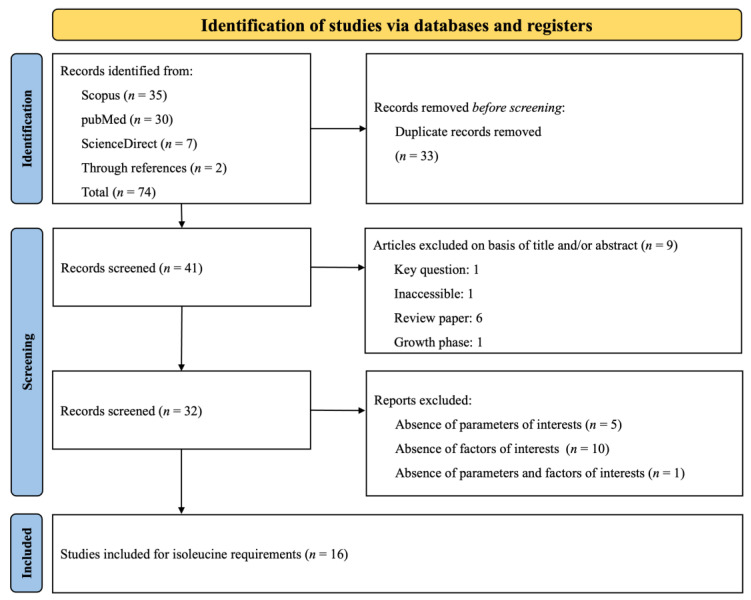
The systematic literature search selection process. The PRISMA diagram details the applied search and selection process for Ile.

The PRISMA flow diagram, presented in Figure 5, summarizes the search strategy employed to investigate Ile requirements. A total of 74 citations were identified across various databases (Scopus: 35, PubMed: 30, ScienceDirect: 7), and 2 additional citations were obtained through reference lists. During the screening process, duplicates and studies that did not meet the specific inclusion criteria were removed. Ultimately, 16 papers were selected for data extraction. Given that some studies included numerous treatments (involving various experiments, basal diets, and phases), these 16 papers were categorized into distinct experiments according to the growth phase (Phase 1: *n* = 24; Phase 2: *n* = 34; Phase 3: *n* = 11). An experiment was defined as the implementation of a basal diet associated with one or more Ile inclusions, expressed as the SID Ile: Lys ratio. The datasets and experimental conditions of the 16 studies, recorded after complete screening, are presented in Table 3. The table lists the authors and year of publication and categorizes each study according to the growth phase of the subjects. It also details the range of the SID Ile: Lys ratio and the range of SID Lys and provides remarks on specific challenge studies. The compilation of research included in the table spans from 2003 to 2022.

#### 3.3.2. Estimation of the SID Ile: Lys Ratio Requirement

**Figure 6:**
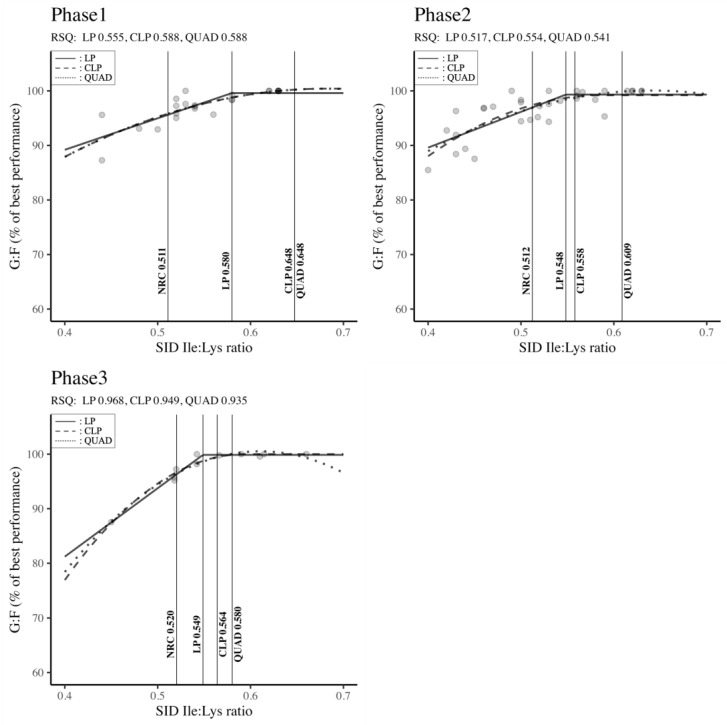
Standardized ileal digestible isoleucine–lysine ratio by body weight classification to maximize feed efficiency in pigs. Phase 1 (BW < 11 kg), Phase 2 (11 kg ≤ BW < 25 kg), Phase 3 (25 kg ≤ BW < 50 kg). RSQ: adjusted R-squared value, LP: linear-plateau model, CLP: curvilinear-plateau model, QUAD: quadratic model. G: F: feed efficiency, SID: standardized ileal digestible.

Figure 6 outlines the SID Ile: Lys ratio requirements across different body weight classifications (Phase 1, Phase 2, and Phase 3) to optimize G: F. No matter the stage of growth, the Ile: Lys ratios derived from the LP, CLP, and QUAD models exceeded the NRC (2012) standards. Specifically, in Phase 1, both the CLP and QUAD models indicated higher Ile: Lys ratio requirements compared to the LP model and NRC recommendations (QUAD: 0.648 = CLP: 0.648 > LP: 0.580 > NRC: 0.511). Similarly, the QUAD model demonstrated the highest requirements, followed by CLP, LP, and NRC in Phase 2 (QUAD: 0.609 > CLP: 0.558 > LP: 0.548 > NRC: 0.512) and Phase 3 (QUAD: 0.580 > CLP: 0.564 > LP: 0.549 > NRC: 0.520). Moderate RSQ values ranging from 0.517 to 0.588 were observed in Phases 1 and 2, while high RSQ values ranging from 0.935 to 0.968 were noted in Phase 3. However, all results were statistically significant (RSQ ≈ 1), affirming that the estimation of SID Ile: Lys ratio requirements was explained well by the independent variables in the model.

### 3.4. Methionine Requirements

#### 3.4.1. Dataset of Methionine

**Figure 7:**
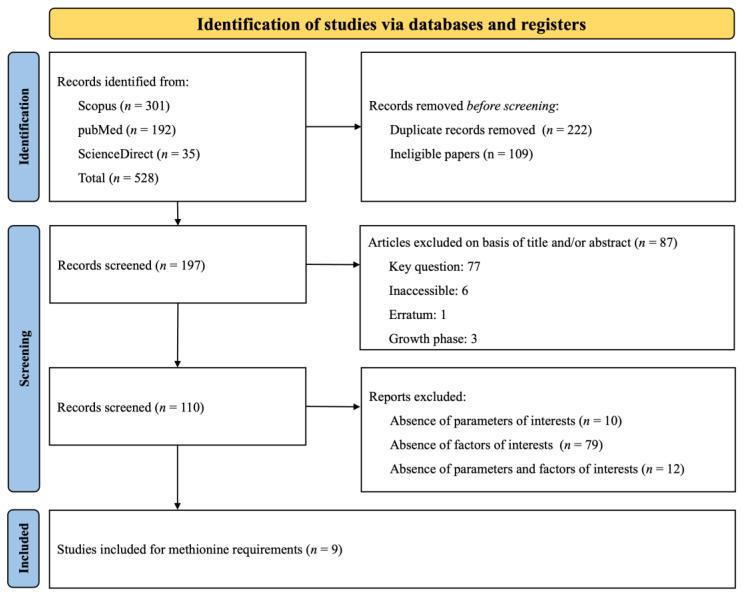
The systematic literature search selection process. The PRISMA diagram details the applied search and selection process for Met.

The PRISMA flow diagram, presented in Figure 7, summarizes the search strategy employed to investigate Met requirements. A total of 528 citations were identified across various databases (Scopus: 301, PubMed: 192, ScienceDirect: 35). During the screening process, duplicates and studies that did not meet the specific inclusion criteria were removed. Ultimately, nine papers were selected for data extraction. Given that some studies included multiple treatments (involving various experiments, basal diets, and phases), these nine papers were categorized into numerous distinct experiments according to the phases studied (Phase 1: *n* = 16; Phase 2: *n* = 13; Phase 3: *n* = 21). An experiment was defined as the implementation of a basal diet associated with one or more Met inclusions, expressed as the SID Met: Lys ratio. The datasets and experimental conditions of the nine studies, recorded after complete screening, are presented in Table 4. The table lists the authors and year of publication and categorizes each study according to the growth phase of the subjects. It also details the range of SID Met: Lys ratio and the range of SID Lys and provides remarks on specific challenge studies. The compilation of research included in the table spans from 2003 to 2022.

#### 3.4.2. Estimation of the SID Met: Lys Ratio Requirement

**Figure 8:**
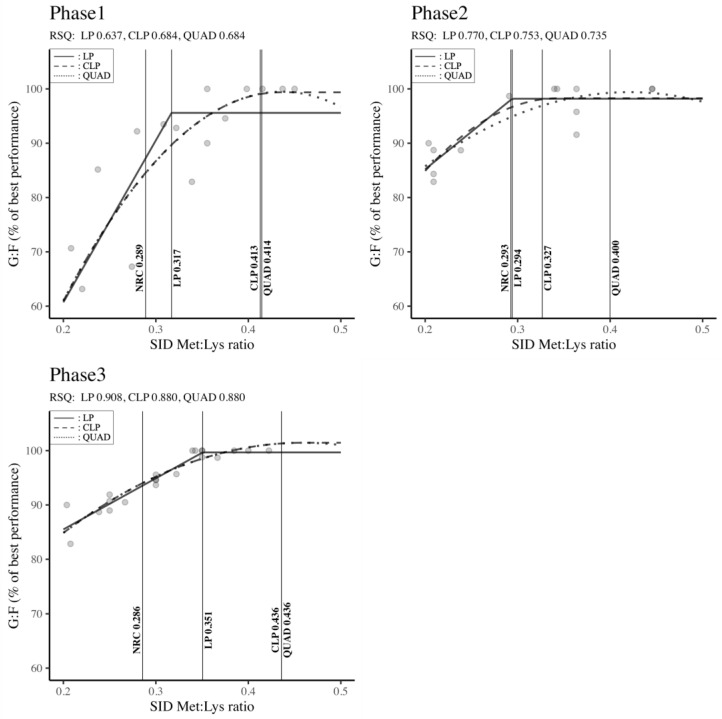
Standardized ileal digestible methionine–lysine ratio by body weight classification to maximize feed efficiency in pigs. Phase 1 (BW < 11 kg), Phase 2 (11 kg ≤ BW < 25 kg), Phase 3 (25 kg ≤ BW < 50 kg). RSQ: adjusted R-squared value, LP: linear-plateau model, CLP: curvilinear-plateau model, QUAD: quadratic model. G: F: feed efficiency, SID: standardized ileal digestible.

The SID Met: Lys ratio requirements across different body weight classifications (Phase 1, Phase 2, and Phase 3) for maximizing G: F are presented in Figure 8. The Met: Lys ratios determined by the LP, CLP, and QUAD models exceeded the NRC (2012) standards, irrespective of the growth phase. In Phase 1 (QUAD: 0.414 > CLP: 0.413 > LP: 0.317 > NRC: 0.289) and Phase 2 (QUAD: 0.400 > CLP: 0.327 > LP: 0.294 > NRC: 0.293), the results depicted in Figure 8 demonstrated that the QUAD model indicated the highest requirements, followed by the CLP, LP, and NRC outputs. For Phase 3, both the CLP and QUAD models, with equal values, emphasized higher requirements in terms of SID Met: Lys ratio compared to the LP model and NRC requirements (QUAD: 0.436 = CLP: 0.436 > LP: 0.351 > NRC: 0.286). In Phase 1, moderate RSQ values ranging between 0.637 and 0.684 were observed, while high RSQ values ranging from 0.735 to 0.908 were noted in Phases 2 and 3. However, all these results were statistically significant (RSQ ≈ 1), confirming that the estimation of SID Met: Lys ratio requirements was accurately explained by the independent variables in the model.

## 4. Discussion

Several statistical models have been developed to determine the requirements of amino acids and proteins in humans [18] and animals, but the choice of method depends essentially on the species of interest, application facilities, and the best fit of the data. This study investigated the amino acid requirements of pigs using the LP, CLP, and QUAD meta-regression models based on numerous outcomes. Indeed, the approximate and controversial results found among individual studies made it difficult to obtain an accurate view of pigs’ amino acid requirements in today’s industry. Therefore, meta-regression analysis, an important instrument to obtain an unbiased evaluation of the available pieces of evidence, is one of the best methods to reach this target. It can accurately summarize the study findings and detect the real effect of an intervention by considering the sources of heterogeneity between articles and increasing the sample size [88]. The great majority of studies use every growth parameter (ADG, BWG, ADFI, and G: F) to estimate the quantity of amino acids maximizing pig performance [11,37,70]. In the present meta-regression analysis, the feed efficiency (G: F) was selected to assess the amino acid requirements in pig nutrition, because it summarized the other growth parameters by expressing the unit of feed consumed per unit of body weight gain.

### 4.1. Tryptophan

It is commonly asserted that Trp, the second or third limiting AA in cereal-based diets for pigs [89], plays an important role in biological functions [13,90] and is involved in the regulation of the immune response, voluntary feed intake, and the synthesis of serotonin [13,14,15]. Hence, it is necessary to determine the Trp requirement of pigs expressed as the ratio of Trp to Lys, with Lys as the first limiting AA to avoid other variables that are similarly restrictive to Trp, thus leading the Trp to Lys ratio to be underestimated [91].

The SID Trp: Lys ratios, according to the LP, CLP and QUAD models, were all superior to NRC [6] requirements regardless of the growth phase. The minimum SID Trp: Lys ratio to support optimum G: F in Phase 1 (QUAD = CLP = 0.229; LP = 0.212), Phase 2 (QUAD = 0.204; CLP = 0.188; LP = 0.183), and Phase 3 (QUAD = 0.207; CLP = 0.199; LP = 0.186) of this meta-regression analysis is on average comparable to a previous meta-analysis investigating the Trp requirements [91] and several original experiments on the same topic [27,36,92]. These results might be due to the improvement of pigs’ growth potential by modern breeding and genetic technologies [28]. Indeed, over 70% of the studies used in the present meta-analysis were conducted from 2012 to 2022, after the release of the NRC requirements (under modern housing conditions and with developed lean-type genetics). In addition, the inclusion of challenged studies in this meta-analysis to assess the Trp: Lys requirements could also be a source of greater values for the optimum inclusion levels of Trp, as inflammatory and stress challenges are known to increase the partitioning of Trp to the immune system [32,33,45,46]. Indeed, during an immune challenge (natural or induced), Trp utilization is increased due to the higher catabolism of Trp via the activation of indoleamine 2, 3 dioxygenases (IDO) [93]. Therefore, during infection, the requirement for Trp is increased. Furthermore, the high Trp: Lys ratio observed in the results of our meta-analysis might be due to the presence of articles in which excess leucine was balanced by a higher incorporation of Trp in pigs’ diets [7]. Numerous authors [94,95,96] suggested that the negative effect of excess leucine could be partially improved by increasing dietary Trp. Based on the previous assertions, we considered that the higher Trp requirements highlighted by our meta-regression are partially attributed to factors such as environmental challenges and diets with excess leucine on the one hand, and the other hand to modern housing and genetic improvement. Moreover, it has been reported that the regression model could have some influence on the estimation of amino acid requirements. Therefore, we would suggest a category of Trp: Lys ratio under challenged conditions, which would be the exact values or range values of the QUAD and CLP models, and another category of Trp: Lys ratio under modern housing and with genetically enhanced pigs represented by the LP model’s values. Hence, the tryptophan requirements for pigs in challenged conditions and the excessive inclusion of leucine would be 0.229, [0188; 0.204], and [0.199; 0.207] in Phases 1, 2, and 3 respectively. The Trp: Lys ratio for genetically improved pigs under modern housing could be 0.212, 0.183, and 0.186 in Phases 1, 2, and 3, respectively. However, deeper studies should be conducted to confirm these results.

### 4.2. Valine

Val belongs to the group of essential branched-chain AAs (BCAAs) that animals cannot synthesize and must therefore be supplied through feeding. In pig nutrition, Val is considered to be the fifth limiting amino acid, and its deficiency is known to decrease the feed intake within 1 h after meal ingestion in the growing stage [16].

The SID Val: Lys ratios determined by the LP, CLP, and QUAD models surpassed the NRC [6] standards in every growth phase (Phase 1, Phase 2, and Phase 3). The Val requirements suggested by the regression models in Phase 1 (QUAD = CLP = 0.717; LP = 0.651), Phase 2 (CLP = 0.695; QUAD = 0.694; LP = 0.662) and Phase 3 (QUAD = CLP = 0.732; LP = 0.690) are comparable to several previous outcomes [50,56,63]. These results might be partially due to the improvement of pigs’ growth potential by modern breeding and genetic technologies [28]. Indeed, over 60% of the studies used in the present meta-analysis were conducted from 2012 to 2022, after the publication of the NRC requirements (under modern housing conditions and with new lean-type genetics). Also, numerous studies [25,56,62,63] with high leucine levels used in this meta-analysis to assess the SID Val: Lys requirements could also be the source of greater values for the optimum inclusion levels of Val. The high incorporation of leucine in those studies was mainly processed through direct amino acid supplementation or the use of a corn co-product (used in 50% of the studies) containing great amounts of Leu. Harper et al. [97] suggested that an oversupply of leucine results in an increase in the requirements of the two other branched-chain amino acids (BCAAs), including Val. Moreover, some authors [16,98] asserted that excess leucine in combination with a low level of Val in the diet had a negative effect on feed intake and consequently on the performance of piglets. According to Nyachoti et al. [99], and Avelar et al. [100], the reduction in feed consumption might also be due to the presence of relatively high fiber in the corn co-product, such as distiller’s dried grains with solubles (DDGS), since fiber increases the bulk volume in the gastrointestinal tract, hence decreasing gut capacity and thereby lowering voluntary feed intake in pigs. Furthermore, with leucine being the most important stimulator of BCAT and BCKD, high levels of leucine can induce the BCAA dehydrogenase complex (BCKDH), an enzyme complex that catalyzes the irreversible degradation of all BCAAs, including Val [96]. Therefore, excess leucine increases Val catabolism, which might become deficient and alter insulin-like growth factor-1 (GH-IGF-1) expression [50]. It has also been reported by Kwon et al. [101] that an over-inclusion of leucine in pigs’ diets reduces protein synthesis, which decreases the availability of Val. Nevertheless, as previously explained, these deficiencies can be overcome by higher inclusions of Val to balance the amino acid requirements in terms of BCAA, as confirmed by several previous experiments [25,102,103].

In addition, the level of Trp in pig diets is also known to influence the valine requirements [11,59] and could explain the higher requirements obtained in our meta-analysis. Following numerous researchers [7,25,104], Clizer et al. [11] suggested that Val and Trp play an integral role in correcting the negative effects of excess dietary leucine when diets contain high inclusion levels of corn co-products.

Apart from the genetic improvement and the modern housing in the pig industry, several other parameters, such as the balance in BCAA and Trp inclusion level, might be the cause of higher Val requirements highlighted by our meta-analysis. Also, the regression model was reported to influence the findings of numerous experiments on amino acid ratios in pig nutrition [56]. Thus, we hypothesize that LP results would be the Val requirements for pigs genetically improved and raised in modern housing, while the Val: Lys ratio of the CLP and QUAD models would be used for pigs fed with diets containing other BCAAs or tryptophan in excess.

Thus, our suggestions for Val requirements in Phases 1, 2, and 3 would be 0.717, [0.694; 0.695], and 0.732, respectively, in diets with excessive BCAAs or Trp inclusion levels, whereas 0.651, 0.662, and 0.690 could be the Val requirements in Phases 1, 2 and 3, respectively, in diets with balanced amino acids, modern housing, and genetically improved pigs.

However, further research using the Val requirements emphasized by our meta-analysis should be conducted to confirm our hypothesis.

### 4.3. Isoleucine

Ile, one of the branched-chain amino acids (BCAAs), is the sixth limiting amino acid in pig nutrition [71]. The SID Ile: Lys ratios derived from the LP, CLP, and QUAD models exceeded the NRC [6] standards regardless of the growth phase. These results might be in part due to the improvement of pigs’ lean meat growth potential by modern breeding and genetic technologies [28]. Indeed, over 80% of the studies used in the present meta-analysis were conducted after the release of NRC results in 2012 (under modern housing conditions and with new pig strains). Also, the presence of studies [72,74] with high leucine or Val levels used in this meta-analysis to assess the SID Ile: Lys requirements could be the source of greater values for the optimum inclusion levels of Ile. Indeed, the Ile requirement increases when other BCAA are in excess, particularly due to an antagonistic effect with excess leucine [105]. This mechanism occurs when elevated leucine increases the levels of the enzyme complex branched-chain keto-acid dehydrogenase, which increases the degradation of all BCAAs; thus, catabolizing Ile and increasing the requirement [95,106]. According to the results of our meta-analysis, the optimum SID Ile: Lys ratio of pigs in Phase 1 varied from 0.580 to 0.648 independent of the model. These higher requirements compared to the NRC recommendations and several articles in the literature review [66,69,70] might be explained by the previous assessment related to genetic improvement and the excessive inclusion of leucine or Val. In Phases 2 and 3, the Ile requirement oscillated from 0.548 to 0.609 and from 0.549 to 0.580, respectively. These results are also higher than several previous experiments [60,67,71,72] on Ile requirements in pig nutrition.

It is noteworthy to mention that the regression model can significantly influence the estimation of amino acid requirements. In their investigation on Val requirements, Liu et al. [56] emphasized a SID Val: Lys ratio of 0.62 using the linear broken-line model, while it was 0.71 when using the quadratic model. Considering the huge number of factors influencing the Ile requirements, we hypothesized that the requirements of QUAD and CLP models would be the right ones for Ile requirements in diets with excess leucine or Val and the LP model values a suggestion of SID Ile: Lys ratio due to genetic improvements in pig balanced diets. Therefore, our recommendation for Ile requirements in Phases 1, 2, and 3 would be 0.648, [0.558; 0.609], and [0.564; 0.580], respectively, in diets with excessive BCAA inclusion levels. On the other hand, 0.580, 0.548, and 0.549 could be the Ile requirements in Phases 1, 2 and 3, respectively, in diets with balanced BCAA inclusion levels.

However, additional research with these estimations of Ile requirements in pig nutrition is necessary to confirm our results.

### 4.4. Methionine

According to Grimble [107], Met, a component of the SAA, is essential not only for protein accretion but also for the production of cytokines, glutathione (GSH), and acute phase proteins (APPs). In addition to its functions in protein synthesis, Met plays key roles in biological functions (e.g., cellular redox function, cell survival and proliferation) and protection against intestinal epithelial growth in young swine [17]. Indeed, a dietary Met deficiency impairs the immune response by affecting the development of lymphoid organs and by reducing the production of antibodies and T-cell proliferation in pigs [108,109] and chickens [110]. Thus, higher SAA supplementation can minimize the negative effects of immunological challenges and help the host maintain health and reduce muscle loss [111]. Met is commonly provided when feeding piglets, because it is the second limiting amino acid in typical diets. The Met: Lys ratios determined by the LP, CLP, and QUAD models exceeded the NRC [6] standards, irrespective of the growth phase. These results could be explained by the improvement of pigs’ growth potential by modern breeding and genetic technologies [28]. Indeed, 100% of the studies used in the present meta-analysis were conducted after the release of the NRC requirements in 2012 (under modern housing conditions and with new lean-type genetics). According to the results of our meta-analysis, the optimum Met: Lys ratio of pigs in Phase 1 varied from 0.317 to 0.417 regardless of the regression model. On the other hand, Rostagno et al. [112], and Sève [113] recommend a Met: Lys of 0.29–0.30 for maximal growth, similar to the NRC [6] report but lower than the present meta-analysis outcomes. The higher Met requirements in Phase 1 observed in our findings compared to previous results might be due to the inclusion of challenged studies [79,80,81] in our meta-analysis. It has been reported that a negative whole-body protein resulting from muscle catabolism to release AA for energy and synthesis of cytokines and APPs may occur when animals are under pathogenic challenge [90,114,115,116]. Therefore, increasing the level of Met in those conditions reduces the adverse effect on pig performance. In Phase 2, the Met requirements in our meta-regression analysis oscillated between 0.294 and 0.400 across the regression models. These results are confirmed by several previous experiments [76,80,85] showing the evolution of Met inclusion level in pig nutrition. The Met requirements in Phase 3 of the present regression meta-analysis ranged from 0.351 to 0.436. The results of Remus et al. [78] and Zhang et al. [83], emphasizing a linear improvement of G: F with a Met: Lys ratio up to 0.50 and 0.38, respectively, broadly support the findings of our study. The reason behind the enhancement of feed efficiency induced by methionine supplementation might be its ability to increase the villus height in the jejunum [84]. In addition, Tsukahara et al. [117] suggested that Met can improve intestinal digestive capacity by increasing the activity of jejunal enzymes.

However, cysteine (Cys) can be converted from Met as needed [118]. Hence, the amount of Met needed in the diet depends on the amount of Cys present.

Based on the different parameters able to influence the Met requirements and the possibility that the regression models could influence the Met: Lys ratio [56], it has been hypothesized that the required levels of Met inclusion in pig diets should be divided into two categories. The first category represents the Met requirements induced by pig genetic improvement and modern housing expressed by the LP model and the methionine requirements in a challenged environment and unbalanced AA highlighted by the QUAD and CLP models of our meta-regression analysis. Hence, the recommendations of our meta-analysis in terms of Met: Lys ratio are described as follows: 0.317, 0.294, and 0.351 for genetically enhanced pigs under modern housing in Phases 1, 2, and 3, respectively, and [0.413;0.414]; [0.327;0.400]; 0.436 for pigs raised under challenged conditions in Phases 1, 2, and 3, respectively.

Nevertheless, additional original experiments using these estimations of Met requirements in pig nutrition are necessary to confirm our results.

## 5. Conclusions

This meta-analysis aimed to assess the requirements for maximizing the feed efficiency of four amino acids (Trp, Val, Ile, and Met) in pig growth phases using the QUAD, CLP, and LP regression models. The results suggested that the requirements of Trp, Val, Ile, and Met in our meta-regression analysis were all higher than the ones in NRC recommendations regardless of the regression models and the growth phases with significant RSQ values (RSQ ≈ 1). Also, the QUAD and CLP regression models emphasized higher requirements than the LP model for the great majority of amino acids and growth phases. The results of the QUAD and CLP models were selected as estimations of amino acid requirements for pigs under challenged conditions and unbalanced diets, whereas the LP model was chosen to estimate the amino acid requirements of genetically improved pigs under a modern housing system. Since the present meta-analysis used a high number of experiments and considered several factors able to influence amino acid requirements, it could be used as a reference for Trp, Val, Ile, and Met inclusion levels in pig nutrition.

## Figures and Tables

**Table 1:** Information on studies used in meta-regression analysis for estimating tryptophan requirements of pigs.

Source	Phase ^1^	Range of SID Trp: Lys Ratio	Range of SID Lys, %	Remarks
Kwon et al. [7]	3	0.18–0.28	1.00	
Tolosa et al. [24]	3	0.16–0.19	0.81–0.89	
Kerkaert et al. [25]	3	0.19–0.21	0.98	
Ma et al. [26]	1	0.15–0.24	1.10	
Capozzalo et al. [27]	1	0.17–0.25	1.23–1.27	
Liu et al. [28]	2–3	0.15–0.23	0.90	
Wensley et al. [29]	2	0.16–0.21	1.25	
Gonçalves et al. [30]	3	0.15–0.25	0.90	
Capozzalo et al. [31]	1	0.16–0.24	1.25	*E. coli* infection
Jayaraman et al. [32]	1	0.16–0.25	1.18	*E. coli* infection
Jayaraman et al. [33]	1	0.19–0.24	1.23–1.33	Unclean condition
Yu et al. [34]	3	0.13–0.21	0.66	
Capozzalo et al. [35]	1	0.16–0.24	1.24	*E. coli* infection
Gonçalves et al. [36]	1–2	0.15–0.25	0.97–1.30	
Nørgaard et al. [37]	1	0.13–0.23	1.09	
Shen et al. [38]	2–3	0.16–1.10	0.90–1.17	Stress
Naatjes et al. [39]	2–3	0.13–0.21	1.05	
Borgesa et al. [40]	1	0.18–0.24	0.92	
Capozzalo et al. [41]	1	0.17–0.26	1.24	*E. coli* infection
Quant et al. [42]	3	0.13–0.18	0.66	
Shen et al. [43]	2	0.21–1.31	0.90	
Zhang et al. [44]	3	0.13–0.25	0.90	
Le Floc’h et al. [45]	1–3	0.15–0.24	1.05–1.22	
Trevisi et al. [46]	1	0.18–0.22	ND ^2^	*E. coli* infection
Eder et al. [47]	3	0.09–0.23	0.87	

^1^ Phase 1 (BW < 11), Phase 2 (11 ≤ BW < 25), Phase 3 (25 ≤ BW< 50). ^2^ No description.

**Table 2:** Information on studies used in meta-regression analysis for estimating valine requirements for pigs.

Source	Phase ^1^	Range of SID Val: Lys Ratio	Range of SID Lys, %	Range of SID Leu: Lys Ratio	Range of SID Ile: Lys Ratio	Remarks
Goodarzi et al. [48]	1–2	0.39–0.75	1.29	0.82	0.29; 0.60	BCAA ^2^
Clizer et al. [11]	3	0.60–0.80	0.98	1.34	0.60	BCAA
Goodarzi et al. [8]	1–2	0.39–0.75	1.29	0.81	0.30; 0.55	BCAA
Habibi et al. [9]	1–2	0.37–0.62	1.29	0.77	0.31; 0.55	BCAA
Kerkaert et al. [25]	3	0.70–0.80	0.98	1.45	0.61	BCAA
Siebert et al. [49]	1–3	0.70–0.76	1.15–1.25	1.03–1.06	0.58	
Millet et al. [50]	1–2	0.58–0.82	1.05	1.05	0.54	
Oliveira et al. [51]	1	0.71–0.87	1.41–1.43	ND ^3^	ND	BCAA
Gonçalves et al. [52]	3	0.57–0.78	0.85	1.54–1.58	0.61–0.62	BCAA
Xu et al. [53]	1–2	0.50–0.80	1.17	0.99	0.53	BCAA
Zhang et al. [54]	2–3	0.45–0.65	1.15	ND	ND	
Clark et al. [55]	1–2	0.50–0.85	1.24	1.10	0.57	
Liu et al. [56]	3	0.55–0.75	0.90	1.13	0.51	BCAA
Soumeh et al. [57]	1–2	0.58–0.78	1.10	ND	ND	
Nemechek et al. [58]	1–2	0.57–0.70	1.30	ND	ND	BCAA
Millet [59]	1–2	0.58–0.67	1.06	0.96	0.52	
Waguespack et al. [60]	2–3	0.61–0.74	0.83	1.30	0.60	
Gaines et al. [61]	2–3	0.55–0.80	1.10	ND	ND	
Gloaguen et al. [62]	2	0.60–0.80	0.95–1.02	1.01–0.69	0.47–0.64	BCAA
Barea et al. [63]	1–2	0.57–0.80	0.92–1.00	ND	0.50–0.60	
Nørgaard and Fernández [64]	1–2	0.60–0.72	1.00	1.02	0.53; 0.62	BCAA
Wiltafsky et al. [65]	1–2	0.49–0.84	0.93–1.02	0.98–1.06	0.59–0.64	

^1^ Phase 1 (BW < 11), Phase 2 (11 ≤ BW < 25), Phase 3 (25 ≤ BW< 50). ^2^ Branched-chain amino acids interaction. ^3^ No description.

**Table 3:** Information on studies used in meta-regression analysis for estimating isoleucine requirements for pigs.

Source	Phase ^1^	Range of SID Ile: Lys Ratio	Range of SID Lys, %	Range of SID Leu: Lys Ratio	Range of SID Val: Lys Ratio	Remarks
Goodarzi et al. [48]	1–2	0.29–0.60	1.29	0.82	0.39–0.75	BCAA ^2^
Clizer et al. [11]	3	0.55–0.75	0.73	1.61	0.78	BCAA
Goodarzi et al. [8]	1–2	0.30–0.55	1.29	0.81	0.39–0.75	BCAA
Habibi et al. [9]	1–2	0.31–0.55	1.29	0.77	0.37–0.62	BCAA
Kerkaert et al. [25]	3	0.61–0.66	0.98	1.45	0.70	BCAA
Clark et al. [66]	1	0.40–0.63	1.24–1.28	1.07–1.09	0.71	
Lazzeri et al. [67]	2–3	0.45–0.73	1.06	0.99	0.65	BCAA
Clark et al. [68]	1–2	0.40–0.63	1.24–1.28	1.07–1.09	0.71	BCAA
Htoo et al. [69]	1–3	0.33–0.70	0.95	1.03–1.33	0.69–0.89	
Soumeh et al. [70]	1–2	0.42–0.62	1.14	ND ^3^	0.70	
Gloaguen et al. [71]	2	0.40–0.55	0.94–0.98	1.01–1.09	ND	BCAA
Nørgaard et al. [72]	1–2	0.42–0.62	1.12	ND	0.70	BCAA
Waguespack et al. [60]	2–3	0.52–0.61	0.83	1.30	0.73	
Barea et al. [73]	2	0.46–0.65	1.00	ND	ND	BCAA
Nørgaard and Fernández [64]	1–2	0.53–0.62	1.00	1.02	0.60–0.72	BCAA
Wiltafsky et al. [74]	1–2	0.36–0.72	0.92–1.02	1.08–1.62	0.68–1.02	BCAA

^1^ Phase 1 (BW < 11), Phase 2 (11 ≤ BW < 25), Phase 3 (25 ≤ BW< 50). ^2^ Branched-chain amino acids interaction. ^3^ No description.

**Table 4:** Information on studies used in meta-regression analysis for estimating methionine requirements for pigs.

Source	Phase ^1^	Range of SID SAA: Lys Ratio	Range of SID Met: Lys Ratio	Range of SID Lys, %	Supplemental Amino Acid ^2^	Remarks
Da Silva et al. [75]	2–3	0.59–0.74	ND ^3^	0.96–1.07	SAA	
Yang et al. [76]	2–3	0.48–0.62	0.20–0.34	1.08	SAA + Met	
Ho et al. [77]	3	0.50–0.70	0.25–0.45	1.00	SAA + Met	
Remus et al. [78]	3	0.37–0.57	0.21–0.39	1.15–1.30	SAA + Met	
Kahindi et al. [79]	1	0.48–0.71	0.22–0.46	1.18	SAA + Met	*E. coli* infection
Zong et al. [10]	1	0.39–0.71	0.20–0.52	1.35	SAA + Met	
Capozzalo et al. [80]	1–2	0.45–0.78	0.21–0.53	1.10–1.20	SAA + Met	*E. coli* infection
Capozzalo et al. [31]	1	0.55–0.66	0.28–0.39	1.25	SAA	*E. coli* infection
Kahindi et al. [81]	1	0.52–0.68	0.24–0.40	1.18	SAA + Met	Unclean condition
Kaewtapee et al. [82]	2–3	0.49–0.69	ND ^3^	1.05	SAA	
Zhang et al. [83]	3	0.50–0.70	0.27–0.47	0.90	SAA + Met	
Chen et al. [84]	1	0.39–0.48	0.20–0.29	1.20	SAA + Met	
Conde-Aguilera et al. [85]	2–3	0.50–0.60	0.24–0.34	1.06–1.09	SAA + Met	
Conde-Aguilera et al. [86]	2	0.38–0.60	0.17–0.40	1.16	SAA + Met	
Frantz et al. [87]	3	0.47–0.63	ND ^3^	1.05	SAA	

^1^ Phase 1 (BW < 11): Phase 2 (11 ≤ BW < 25), Phase 3 (25 ≤ BW< 50). ^2^ SAA, supplementation with sulfur amino acids; SAA + MET, supplementation with methionine without concurrent increase in cysteine. ^3^ No description.

## Data Availability

The original contributions presented in this study are included in the article; further inquiries can be directed to the corresponding author.
